# Supplementary material for: Comprehensive Genetic Analysis of Druze Provides Insights into Carrier Screening
Source: Genes (Basel). 2023 Apr 18;14(4):937. doi: 10.3390/genes14040937 (PMC10137689; doi:10.3390/genes14040937)
Supplement: Supplementary file 1 [file genes-14-00937-s001.zip › genes-2260434-supplementary.pdf]

Table S1. Potentially clinically relevant genes and variants from the exome sequencing analysis which were previously associated with the Druze population.

Rs Number	ClinVar ID [a]	Condition [b]	Gene (OMIM#)	Nucleotide Alteration (Amino Acid)	Mutation type	Location [c]	Durze AF (AC/AN)	Heterozygous/ Homozygous	General Population AF (AC/AN)	Fisher's p-value	Middle East AF (AC/AN)
rs104894176	13709 (2, P)	Familial hemophagocytic lymphohistiocytosis 2 (AR)	PRF1 (170280)	NC_000010.11:g.70598599C>T	Nonsense	10 - 70598599 C > T	0.004 (1/236)	1/0	2.6e-5 (4/152194)	0.008	0 (0/316)
rs781266802	189015 (2, P/LP)	Wilson's disease (AR)	ATP7B (277900)	NM_000053.4:c.3649_3654del (NP_000044.2:p.Val1217_Leu1218 del)	Inframe deletion	13 - 51939095 TCAGAAC > T	0.004 (1/236)	1/0	1.3e-05 (2/152192)	0.005	0 (0/316)
rs104894318	3802 (2, P)	Tyrosinase-negative oculocutaneous albinism (AR)	TYR (606933)	NM_000372.5:c.1342G>A (NP_000363.1:p.Asp448Asn)	Missense	11 - 89284930 G > A	0.004 (1/236)	1/0	7.6e-06 (2/264690)	0.003	NA
rs756959430	437454 (2, P)	Mucolipidosis III Gamma (AR)	GNPTG (252605)	NM_032520.5:c.499dupC (NP_115909.1:p.Leu167fs)	Frameshift	16 - 1362287 A > AC	0.008 (2/236)	2/0	3.3e-5 (5/151942)	5e-5	0 (0/316)
rs587779815	127337 (2, P/LP)	Ataxia-telangiectasia syndrome (AR)	ATM (208900)	NM_000051.4:c.1339C>T (NP_000042.3:p.Arg447Ter)	Nonsense	11 - 108250804 C > T	0.008 (2/236)	2/0	1.3e-5 (2/151854)	1.4e-5	0 (0/316)
rs1370579526	559417 (1, P)	Combined oxidative phosphorylation deficiency 42(AR)	GATC (617210)	NM_176818.3:c.233T>G (NP_789788.1:p.Met78Arg)	Missense	12 - 120446808 T > G	0.008 (2/236)	2/0	4e-6 (1/247366)	2.7e-6	NA

rs121965022	11914 (2, P)	Mucopolysaccharidosis type I (AR)	IDUA (252800)	NM_000203.5:c.192C>A (NP_000194.2:p.Tyr64Ter)	Nonsense	4 - 987842 C > A	0.01 (3/236)	3/0	1.3e-05 (2/152204)	3.7e-8	0 (0/316)
rs1555547112	520436 (1, P)	Nonsyndromic hearing loss 3 (AR)	MYO15A (602666)	NM_016239.4:c.9083+6T>A	Intron	17 - 18158644 T > A	0.02 (4/236)	4/0	NA	NA	NA
rs28940579	2540 (2, P/LP)	Familial Mediterranean fever (AR)	MEFV (608107)	NC_000016.10:g.3243310A>G	Missense	16 - 3243310 A > G	0.02 (5/236)	5/0	0.001 (219/152066)	2.9e-5	0.01 (4/316)
rs397509360	29 (2, P)	Primary hyperoxaluria, type 3 (AR)	HOGA1 (613597)	NM_138413.4:c.938AGG[2] (NP_612422.2:p.Glu315del)	Inframe deletion	10- 97611611 TGAG > T	0.03(6/236)	6/0	0.0003 (41/152222)	1.31e-10	0 (316/0)

a Number of stars and pathogenic level (A- Association, P- Pathogenic or LP- Likely Pathogenic, VUS- variant of uncertain significance) as labeled by Clinvar. b Autosomal Dominant= AD ; Autosomal Recessive= AR ; X-linked Dominant= XLD ; Multifactorial= M. c Chromosome - Position Reference > Alternative. d Variant in a low complexity region according to GnomAD.

Table S2. Potentially clinically relevant genes and variants from the HGDP genome sequencing analysis which were previously associated with the Druze population.

Rs Number	ClinVar ID [a]	Condition [b]	Gene (OMIM#)	Nucleotide Alteration (Amino Acid)	Mutation type	Location [c]	Durze AF (AC/AN)	Heterozygous/ Homozygous	General Population AF (AC/AN)	Fisher's p-value	Middle East AF (AC/AN)
rs62638191	8003(2, P/LP)	Pigmentary retinal dystrophy (AR/AD)	RDH5 (601617)	NM_002905.5:c.712G>T (NP_002896.2:p.Gly238Trp)	Missense	12- 55724028 G > T	0.01 (1/80)	1/0	0.0002 (30/152092)	0.02	0.003 (1/316)
rs80338940	17029 (2, P)	Deafness, type 1A (AR)	GJB2 (121011)	NC_000013.11:g.20192782C>T	Splice donor	13 - 20192782 C > T	0.01 (1/80)	1/0	0.0003 (42/152092)	0.02	0.003 (1/316)
rs28940578	2539 (1, P/VUS)	Familial Mediterranean fever (AR)	MEFV (608107)	NC_000016.10:g.3243405C>T	Missense	16 - 3243405 C > T	0.01 (1/80)	1/0	7.9e-05 (12/152204)	0.007	0.003 (1/316)
rs28936701	7733 (2, P)	Glaucoma 3A (AR)	CYP1B1 (601771)	NC_000002.12:g.38070949G>A	Missense	2 - 38070949 G > A	0.02 (2/80)	2/0	5.3e-05 (8/152118)	1.2e-5	0.003(3/316)

a Number of stars and pathogenic level (A- Association, P- Pathogenic or LP- Likely Pathogenic, VUS- variant of uncertain significance) as labeled by Clinvar. b Autosomal Dominant= AD ; Autosomal Recessive= AR ; X-linked Dominant= XLD ; Multifactorial= M. c Chromosome - Position Reference > Alternative. d Variant in a low complexity region according to GnomAD.
